# Preparation of novel (-)-gossypol nanoparticles and the effect on growth inhibition in human prostate cancer PC-3 cells *in vitro*

**DOI:** 10.3892/etm.2015.2172

**Published:** 2015-01-08

**Authors:** CAI-LING JIN, MEI-LING CHEN, YING WANG, XIAO-CHUN KANG, GUANG-YE HAN, SU-LING XU

**Affiliations:** 1 Department of Oncology, The First Affiliated Hospital of Xinxiang Medical University, Weihui, Henan 453100, P.R. China; 2 Department of Urology, The First Affiliated Hospital of Xinxiang Medical University, Weihui, Henan 453100, P.R. China

**Keywords:** (-)-gossypol, nanoparticle, apoptosis, prostate cancer

## Abstract

The aim of the present study was to investigate the antitumor effects and possible mechanism of (-)-gossypol nanoparticles, loaded with vv polyethylene glycol-maleimide (mPEG-Mal), *in vitro*. Emulsification-volatilization was used to prepare the loaded (-)-gossypol nanoparticles. The toxicity of blank nanoparticles on human prostate cancer PC-3 cells and human prostate RWPE-1 cells was measured. The antitumor effects of the nanoparticles on PC-3 cells were evaluated by an MTT assay, acridine orange staining and transmission electron microscopy *in vitro*, and the results were compared with those of free (-)-gossypol. In addition, the mRNA expression levels of Bcl-2 and Bak were measured using semi-quantitative reverse transcription polymerase chain reaction. The growth inhibition activity of the loaded (-)-gossypol nanoparticles was found to be dose- and time-dependent, and similar to the activity of free (-)-gossypol. The nanoparticles induced apoptotic morphological changes on the PC-3 cells, downregulating the mRNA expression level of Bcl-2 and upregulating the mRNA expression level of Bak. Blank nanoparticles exhibited no evident toxicity on PC-3 and RWPE-1 cells at a high dose. Therefore, the mPEG-Mal loaded (-)-gossypol nanoparticles demonstrated a favorable antitumor activity and no toxicity. The nanoparticles were able to induce the apoptosis of prostate cancer cells; thus, may be a potential antitumor nanodrug.

## Introduction

Gossypol is a phenolic aldehyde extracted from cotton and tropical plants that is able to permeate cells. It forms an extensive network of hydrogen bonding with residues Arg146 and Asn143 in Bcl-2 throughout the aldehyde group and the adjacent hydroxyl group on the right naphthalene ring (1). Gossypol is as a Bcl-2 homology domain 3 (BH3)-mimetic inhibitor of antiapoptotic Bcl-2 family members, including Bcl-2, Bcl-xL and Mcl-1, and induces apoptosis in various types of cancer (2–4). Gossypol also mediates a number of signaling pathways, including inhibiting the growth of prostate cancer cells by modulation of the TGF-β/Akt signaling pathway (5) and activation of TP53 (6), and enhancement of radiation-induced apoptosis through the SAPK/JNK pathway (7).

(-)-Gossypol, an optical isomer of gossypol, was found to significantly inhibit the growth of various tumor cells. For example, (-)-gossypol has been shown to inhibit the expression of antiapoptotic proteins, including Bcl-2, Bcl-xL and Mcl-1, and further induce the expression of apoptosis-associated proteins, such as Noxa, Puma and Bim, thereby inducing cell apoptosis (8,9). *In vivo* studies have demonstrated that (-)-gossypol presents good antitumor activity in lymphoma, head and neck tumors (4,10,11). However, (-)-gossypol has not be used as an antitumor agent due to a number of limitations, including poor water solubility, single-route drug administration and low bioavailability. In addition, at high concentrations, (-)-gossypol may be highly toxic to the liver and intestinal tract (12).

In order to improve the application of gossypol as an antitumor agent, the polymer carrier, methoxy polyethylene glycol-maleimide (mPEG-Mal), was loaded on (-)-gossypol nanoparticles using an emulsification-volatilization method. The aim of the present study was to further investigate the toxicity of the mPEG-Mal polymer carrier and the antitumor effect of (-)-gossypol nanoparticles.

## Materials and methods

### Cell lines and reagents

Human prostate RWPE-1 and prostate cancer PC-3 cell lines were obtained from the Animal Experiment Center of the Fourth Military Medical University (Xi’an, China), and (-)-gossypol was obtained from the College of Life Science of Xi’an Jiaotong University (Xi’an, China). Written informed consent was obtained from the patient prior to this. The polymer carrier, mPEG-Mal (5,000 D; Beijing Kaizheng Biotech Development Co., Ltd., Beijing, China), MTT dye (Shanghai Sangon Biotech Co., Ltd., Shanghai, China) and acridine orange (AO) dye (One Lambda, Beijing China) were purchased for the purpose of the experiments. Reverse transcription polymerase chain reaction (RT-PCR) primers were synthesized by Shanghai Sangon Biotech Co., Ltd.

### Main instruments

The following instruments were used in the experiments: NuAire AutoFlow CO_2_ cell incubator (NuAire, Plymouth, MN, USA); PCR EDC-810 amplifier (Dongsheng Biotech Co., Ltd., Beijing, China); multifunctional gel imaging system (GL2200; Kodak, Rochester, NY, USA); JEM-2000EX transmission electron microscope (Electronic optical Company, Osaka, Japan); and BX60 inverted fluorescence microscope (Olympus Corporation, Tokyo, Japan).

### Preparation of the mPEG-Mal nanoparticles and their main features

An emulsification-volatilization method was used to prepare the loaded (-)-gossypol nanoparticles. Blank nanoparticles were also prepared using the same method, after which they were frozen. The average diameter of the nanoparticles was 65.1 nm, the (-)-gossypol-loading efficiency was 97.5±1.57% and the loading capacity was 37.5±0.27%. *In vitro* release experiments demonstrated that the (-)-gossypol nanoparticles had controlled-release characteristics.

### In vitro detection of the toxicity of (-)-gossypol nanoparticles using an MTT assay

PC-3 cells were adjusted to a concentration of 5×10^6^ cells/ml and inoculated in 96-well culture plates, with each well holding up to a volume of 100 μl. Free (-)-gossypol or (-)-gossypol nanoparticles at different concentrations were added to a plate (one plate for each concentration of gossypol nanoparticles), and the final concentrations in the wells were 2.5, 5, 10 or 20 μg/ml. Each well was followed by three duplicate wells. After 48 h, 20 μl MTT (5 mg/ml) was added and the plates were cultured for 4 h. The culture solution was centrifugally removed. Next, 150 μl DMSO was added to each well and the plate was vortexed for 10 min until the crystals were fully dissolved. ELISA was used to detect the absorbance (optical density) at a wavelength of 490 nm, and the median inhibitory concentration (IC_50_) was calculated. The same method was used to measure the growth inhibition of the blank nanoparticles (control sample) on the PC-3 and RWPE-1 cells, in order to assess the toxicity of the polymer carrier.

### AO staining

PC-3 cells were inoculated in 96-well plates, with each well containing 100 μl cell suspension. In each well, 100-μl samples of the different (-)-gossypol nanoparticle concentrations were added. For the control group, 100 μl RPMI-1640 culture medium (ScienCell, Carlsbad, CA, USA), supplemented with 10% fetal bovine serum (Gibco^®^, Invitrogen Life Technologies, Grand Island, NY, USA), was added. After 48 h, 10 μl AO dye (Bio-Teck, Beijing, China) was added to each well and cultured for 15 min. The morphological changes were observed under an inverted fluorescence microscope (Olympus Corporation). Each sample was found to contain ≥100 cells, and the percentage of apoptotic cells was calculated.

### Cellular ultrastructure observations

Cells were inoculated in culture bottles at a concentration of 2×10^5^ cells/ml, with each bottle containing 4 ml cell suspension. After 24 h, 4 ml (-)-gossypol nanoparticles, at a concentration of 10 μg/ml, was added to each bottle. For the control, 4 ml DMSO (Beyotime, Hangzhou, China) at the same concentration was added to the RPMI-1640 culture medium. After 48 h, trypsin was used to digest and wash any unreacted RPMI-1640 culture medium, and the solution was centrifugally subsided. Next, 4% glutaraldehyde (Huakang, Suzhou, China) was added and incubated for 2 h, which was followed by two washes with phosphate-buffered saline. Osmic acid (1%) that had been precooled at 4°C was added, and after 1 h, the samples were dehydrated, embedded in paraffin and cut into 70-nm segments. Uranyl acetate and citrate staining were used to dye the samples, and their cellular morphology was observed under a transmission electron microscope.

### Semi-quantitative RT-PCR detection of Bcl-2 and Bak mRNA expression

The concentration of the cells was adjusted to 5×10^5^ cells/ml. A total of 2 ml cells was added to a cell culture bottle. Samples from the control group and the experimental group (containing 10.0 μg/ml (-)-gossypol nanoparticles) were placed into the cell culture bottle; 8 ml cell culture fluid was added and cultured for 48 h. The primer design and the experimental methods followed in these experiments were based on the methods of a previous study (13).

### Statistical analysis

SPSS 10.0 software (SPSS, Inc., Chicago, IL, USA) was used to conduct statistical analysis. χ^2^ analysis and the t-test were used to evaluate the results, where P<0.05 was considered to a indicate statistically significant difference.

## Results

### Effect of (-)-gossypol nanoparticles on the proliferation of PC-3 cells

**Figure 1:**
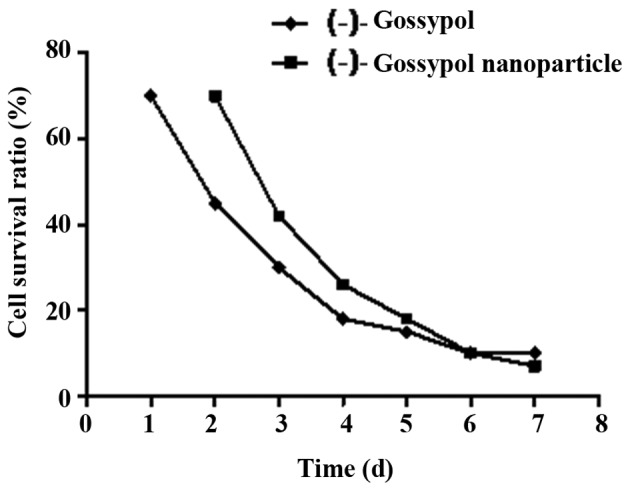
Effect of (-)-gossypol nanoparticles and free (-)-gossypol on the growth of PC-3 cells.

**Figure 2:**
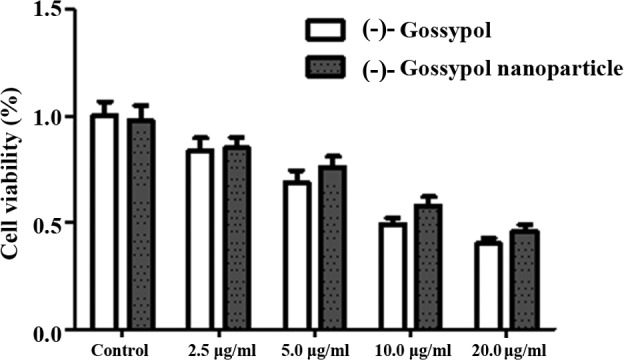
Effect of (-)-gossypol nanoparticles and free (-)-gossypol at different concentrations on the growth of PC-3 cells.

When the (-)-gossypol nanoparticles and free (-)-gossypol reached a concentration of 10.0 μg/ml, they demonstrated evident antitumor activity against prostate cancer PC-3 cells *in vitro* (Fig. 1). As shown in Fig. 1, the inhibition effects of (-)-gossypol nanoparticles and free (-)-gossypol on the proliferation of PC-3 cells increased with time. In addition, following culture for 72 h, the inhibition effects of (-)-gossypol nanoparticles and free (-)-gossypol on the proliferation of PC-3 cells increased with increasing concentration (Fig. 2). At the various time points, the IC_50_ of the (-)-gossypol nanoparticles was slightly higher compared with the free (-)-gossypol; however, no statistically significant difference was observed (P>0.05).

### Toxicity assessment of the blank carrier

Following the addition of the blank carrier in the PC-3 and RWPE-1 cells for 48 h, no evident change was observed with regard to the survival rate of the cells. When the concentration of the blank carrier reached 200 μg/ml, the survival rate of the PC-3 and RWPE-1 cells decreased; however, the rate remained >95%, and no evident change in the cell shape was observed (data not shown).

### AO staining results

**Figure 3:**
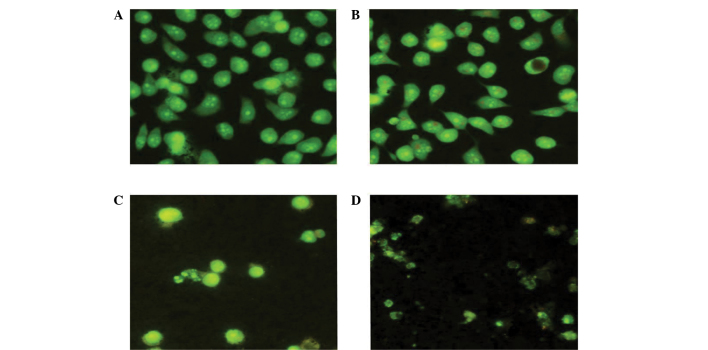
PC-3 cells following acridine orange staining (magnification, ×400). (A) Normal PC-3 cells cultured for 48 h; (B) PC-3 cells after culturing with 5.0 μg/ml (-)-gossypol nanoparticles for 48 h; (C) PC-3 cells after culturing with 10.0 μg/ml (-)-gossypol nanoparticles for 48 h; and (D) PC-3 cells after culturing with 20.0 μg/ml (-)-gossypol nanoparticles for 48 h.

After culturing the control group cells for 48 h, the cells were concentrated together and the cell chromatin was evenly distributed (Fig. 3). Upon culturing with 5.0 μg/ml (-)-gossypol nanoparticles for 48 h, only part of the cell nucleus was pyknotic and cell apoptosis was observed. Following culture with 10.0 μg/ml (-)-gossypol nanoparticles for 48 h, the number of apoptotic cells was markedly increased, the cell chromatin was not evenly distributed and a number of cells had burst. After culturing with 20.0 μg/ml (-)-gossypol nanoparticles for 48 h, the number of cells decreased, cell apoptosis was evident, the chromatin was arranged along the nuclear membrane in a crescent-shape and cell fragmentation was observed.

### Changes in the cellular ultrastructure

**Figure 4:**
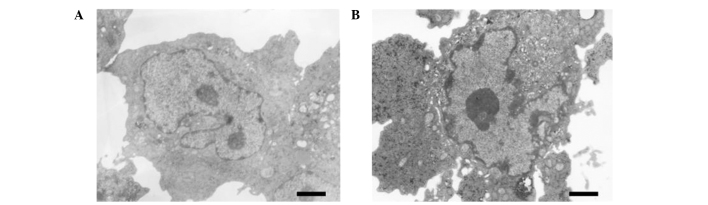
Ultrastructure of PC-3 cells. (A) Normal PC-3 cells and (B) PC-3 cells cultured with 10.0 μg/ml (-)-gossypol nanoparticles for 48 h (scale bar,2 μm).

In the normal PC-3 cells, microvilli were detected on the surface, small fat droplets and lipofuscin particles were observed, and the nucleus chromatin was shown to mainly consist of euchromatin (Fig. 4A). Following culture with 10.0 μg/ml (-)-gossypol for 48 h, the PC-3 cells presented typical features of apoptotic cells, including the disappearance of microvilli from the cell surface, smooth edges and agglutinated nuclear chromatin that was arranged close to the edge of the nuclear membrane (Fig. 4B).

### Semi-quantitative RT-PCR detection of Bcl-2 and Bak mRNA expression levels

Through semi-quantitative RT-PCR detection, the size of the Bcl-2, Bak and GAPDH genes were determined as 387, 360 and 142 bp, respectively, consistent with the expected values. Following culture with 10.0 μg/ml (-)-gossypol nanoparticles for 48 h, the mRNA expression levels of Bcl-2 were downregulated in the PC-3 cells, and the Bcl-2/GAPDH ratio decreased from 0.17 to 0.08. In addition, the mRNA expression levels of Bak were upregulated, and the Bak/GAPDH ratio increased from 0.62 to 0.89.

## Discussion

As traditional *in vivo* medicine drug carriers, nanomaterials have become increasingly important in modern medicine and possess a good application potential (14,15). PEG is a synthetic polymer material, which can dissolve in water and is soluble in certain organic solvents. PEG is the only polymer material approved by the US Food and Drug Administration for use in food and pharmaceuticals (16). Coating lipophilic drugs with PEG may improve their solubility and stability, while reducing or eliminating the body’s rejection of the drug effects, and lowering the rate of drug metabolism. In addition, coating drugs with PEG can extend the cycling time of the drug, improve distribution in the body and diminish any adverse reactions (17). mPEG-Mal is a modified PEG polymer material with good biodegradability and biocompatibility. In addition, the maleimide functional group allows the polymer to chemically connect with proteins containing a sulfhydryl group or with antibodies at room temperature. The mild reaction conditions do not destroy the proteins and the activity of antibodies, providing the necessary structural basis for future research on nanoactive targeting drugs.

In the present study, (-)-gossypol nanoparticles were shown to effectively inhibit the growth of prostate cancer PC-3 cells *in vitro*, with their toxicity similar to that of free (-)-gossypol. In a previous study (18), the slow-release ability of (-)-gossypol was revealed, with a potential release of ~40% in 48 h. At the same dose, (-)-gossypol nanoparticles release less compared with free (-)-gossypol, indicating that the antitumor effect of (-)-gossypol nanoparticles is stronger than that of free (-)-gossypol. This may result from the ability of nanoparticles to penetrate into cells through cell endocytosis, which is not possible for small-molecule drugs (19,20).

With regard to the mechanisms, (-)-gossypol nanoparticles can function as micromolecule inhibitors to inhibit the expression of the anti-apoptotic protein Bcl-2 (21). In the present study, apoptosis was induced in prostate cancer cells and the mechanism was similar to that observed in previous studies (8,9), indicating that the preparation of nanoparticles has no effect on the biological activity and molecular structure of (-)-gossypol. The preparation process was simple and the reaction conditions were mild. In addition, the blank carrier was found to be safe and non-toxic; thus, demonstrated good application potential. Tumor-bearing animal models should be used in future studies to further investigate the antitumor effects and pharmacokinetic properties of nanoparticles, after which active targeting studies may be performed.
